# An Assessment of Dementia Caregivers’ Interaction With Community-Based Services

**DOI:** 10.1093/geroni/igab046.2819

**Published:** 2021-12-17

**Authors:** Deepika Pugalenthi Saravanan, Nihal Satyadev, Natashia Townsend, Katherine Rose, Harrison Ma, Donna Benton, Lené Levy-Storms

**Affiliations:** 1 UCLA, San Ramon, California, United States; 2 The Youth Movement Against Alzheimer's, Los Angeles, California, United States; 3 Youth Movement Against Alzheimer’s, Culver City, California, United States; 4 Youth Movement Against Alzheimer's, Los Angeles, California, United States; 5 University of Southern California, Los Angeles, California, United States; 6 University of Southern California Family Caregiver Support Center, Los Angeles, California, United States; 7 UCLA Luskin School of Public Affairs/Social Welfare, West Hills, California, United States

## Abstract

Respite care is an important service to address caregivers' stress and fatigue when caring for a person with dementia (PWD). YouthCare is a non-medical, at-home, intergenerational respite care program that partners trained student volunteers with PWDs. The Family Caregiver Survey was created and distributed to caregivers of PWDs in Los Angeles to better understand interactions with the community and its caregiver services. The survey assesses caregivers’ demographics, daily activities, mental health, and the type of respite support needed. The survey findings (n=47) show that 53.2% of caregivers are 54 and older and 83% females. 40.4% of the caregivers listened to the radio primarily in the morning while 61.7% watched television in the afternoon to evening time. For transportation of PWDs to and from destinations, 78.3% of caregivers reported using their own vehicles. In regards to their mental health, 61.7% of the caregivers stated that they felt tired and unmotivated to complete daily activities. When asked why they sought respite services, 40% stated that they were overwhelmed by the responsibilities in addition to their own work. The groups that primarily support caregivers are family and professional respite services. Findings indicate that caregivers are most likely to trust resource recommendations from family and friends. Similar surveys should be administered in other cities and in rural locations to improve the generalizability of our findings.

